# Case Report: Laparoscopy-assisted trans-umbilical Meckel's diverticulectomy for Meckel's diverticulum complicated by internal hernia: a three-case series and narrative literature review

**DOI:** 10.3389/fped.2026.1752428

**Published:** 2026-04-07

**Authors:** Qiang Hao, Lu Han, Wenrui Mi, Tingting Hu, Aiqin Lin, Jie Liu

**Affiliations:** 1 Department of Pediatric Surgery, The First Affiliated Hospital of Wannan Medical College (Yijishan Hospital of Wannan Medical College), Wannan Medical College, Wuhu, China; 2 Department of Medical Biology of Wannan Medical College, Wannan Medical College, Wuhu, China; 3 Department of Graduate School, Wannan Medical College, Wuhu, China; 4 Department of Science and Technology, The First Affiliated Hospital of Wannan Medical College (Yijishan Hospital of Wannan Medical College), Wannan Medical College, Wuhu, China

**Keywords:** hernia, internal, LATUM, Meckel's diverticulum, pediatric

## Abstract

**Background:**

Meckel's diverticulum (MD) is the most prevalent congenital anomaly of the small intestine. Internal hernia secondary to MD is extremely rare in children and poses a diagnostic and therapeutic challenge.

**Case presentation:**

We report three consecutive paediatric cases successfully managed by laparoscopy-assisted trans-umbilical Meckel's diverticulectomy (LATUM) and provide a comprehensive review of the world literature. Clinical data of three children with MD complicated by internal hernia treated between March 2023 and April 2024 were retrospectively analysed. A systematic search of PubMed, Web of Science and Google (up to June 2025) was performed to identify previously published paediatric cases. Two boys and one girl (median age: 7 years, range: 7–11) presented with acute abdominal pain and vomiting. Pre-operative imaging suggested intestinal obstruction or intussusception. Laparoscopy revealed internal hernia formed by a mesodiverticular band (MDB) in all cases. LATUM was completed without conversion. Median operative time was 65 min (range: 50–90, IQR: 55–75), estimated blood loss was 10 mL (range: 5–15, IQR: 7.5–12.5), and time to first flatus was 24 h (range 18–36, IQR 20–30). Median hospital stay was 7.5 days (7–8). No complications occurred during a median follow-up of 16 months. The literature review yielded 25 additional paediatric cases. Including our series, 28 children have been reported; 9 cases (32.14%) used LATUM, 8 cases (28.57%) used open resection, and 11 cases (39.29%) did not describe the surgical approach.

**Conclusion:**

LATUM offers a safe, minimally invasive and cosmetically superior option for children with MD complicated by internal hernia. A high index of suspicion and early laparoscopy are crucial to avoid bowel necrosis.

## Background

Meckel's diverticulum (MD), a persistent remnant of the vitelline duct, represents the most prevalent congenital anomaly of the gastrointestinal tract, affecting approximately 2% of the general population with a male predominance (1). While the majority of cases remain asymptomatic throughout life, 4%–7% of individuals develop complications including gastrointestinal bleeding, intussusception, perforation, intestinal obstruction, or diverticulitis (2). Among these complications, internal hernia secondary to mesodiverticular bands (MDB) or vitelline vessel remnants is exceptionally rare, accounting for less than 1% of all MD-related morbidities, yet carries significant risk of bowel incarceration, strangulation, and potential necrosis if not promptly recognized and treated (3).

Laparoscopy-assisted trans-umbilical Meckel's diverticulectomy (LATUM) represents an innovative hybrid approach that strategically combines the diagnostic and therapeutic advantages of minimally invasive surgery with the precision of conventional open techniques (3). The fundamental principle of LATUM involves initial diagnostic laparoscopy to comprehensively evaluate the entire small bowel, identify the anatomical abnormality, and assess bowel viability, followed by exteriorization of the affected segment through a small extension of the umbilical incision for extracorporeal resection and anastomosis (1). This approach offers distinct technical advantages: the laparoscopic component enables magnified visualization of the peritoneal cavity and precise identification of the MDB without the need for multiple incisions, while the extracorporeal component allows for tactile feedback, accurate wedge resection of the diverticulum, and secure hand-sewn anastomosis under direct vision—particularly important in pediatric patients where stapling devices may be oversized. The single-incision trans-umbilical approach provides superior cosmetic outcomes by concealing the surgical scar within the natural umbilical folds, minimizing the psychological impact on pediatric patients and reducing the risk of postoperative adhesive bowel obstruction compared to multi-port laparoscopy or open surgery (4). Furthermore, extracorporeal anastomosis has been demonstrated to be safe and effective in children, with comparable leak rates to intracorporeal techniques while reducing operative time and technical complexity (5, 6).

We describe three consecutive children managed with LATUM and summarise the world experience. We retrospectively analyzed all children who underwent Meckel's diverticulum surgery with internal hernia at Yijishan Hospital of Wannan Medical College between March 2023 and April 2024. Demographics, clinical manifestations, imaging, surgical details, pathology, and follow-up were extracted from electronic records. The studies involving human participants were reviewed and approved by Ethics Committee of Yijishan Hospital of Wannan Medical College (No. 2025-218), and written consent was obtained from the parents of the children.

## Case presentation

### Case 1

**Figure 1:**
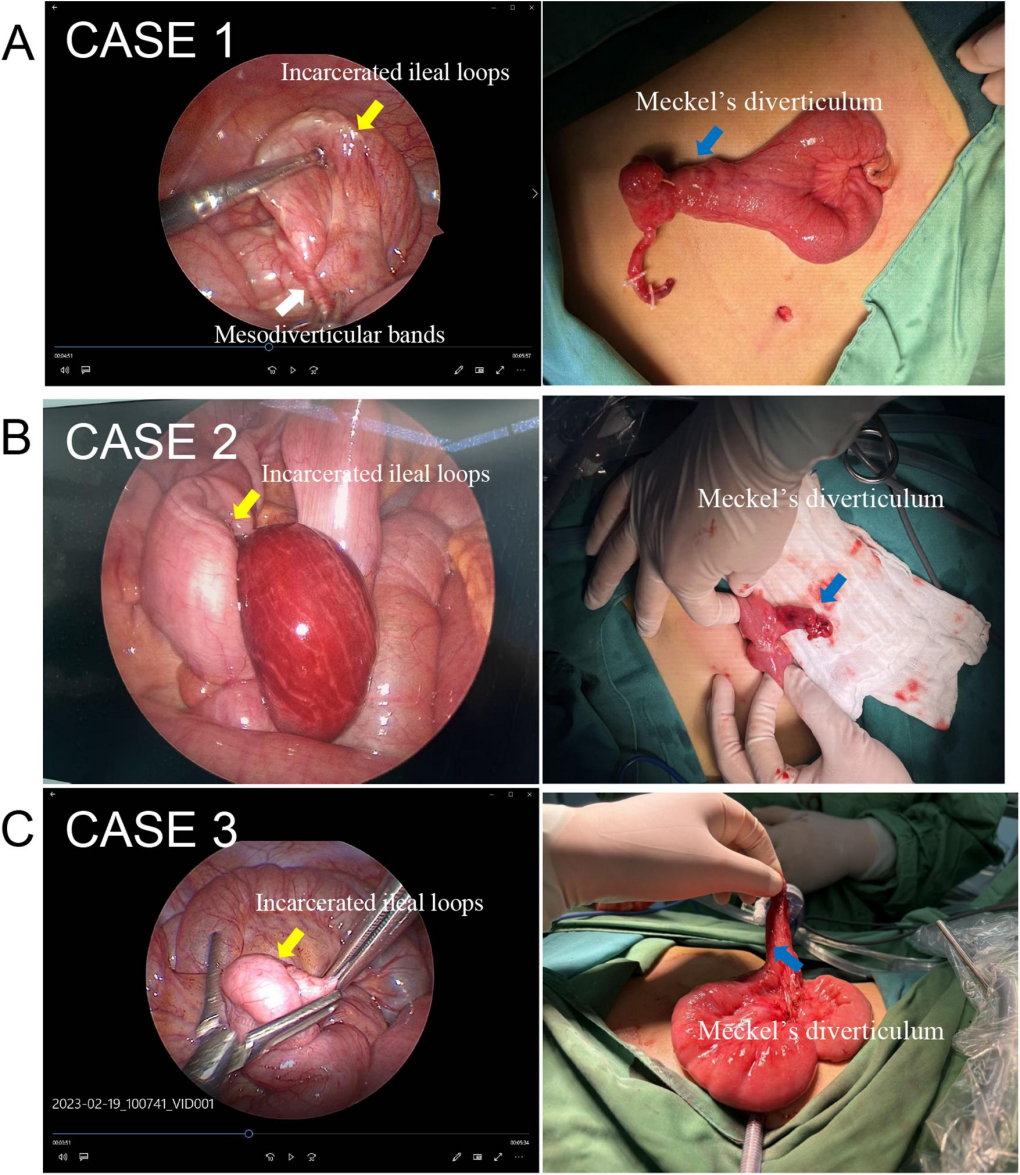
Intraoperative screenshots of three pediatric patients with Meckel's diverticulum and internal hernia in this study. Panel **A** shows two images from Case 1: the left image is a laparoscopic view highlighting incarcerated ilealloops and mesodiverticular bands, while the right shows Meckel's diverticulum exposed on a patient's abdomen. Panel **B** includes Case 2: the left image presents a laparoscopic view of incarcerated ileal loops, and the right image displays Meckel's diverticulum being held by gloved hands. Panel **C** contains Case 3: the left image is a laparoscopic view of incarcerated ileal loops, and the right image shows Meckel's diverticulum extracted and held above the surgical field. Each case demonstrates surgical identification and handling of Meckel's diverticulum and associated intestinal loops.

A 7-year-old boy presented with abdominal pain and bilious vomiting for 48 h. Abdominal CT demonstrated distal small-bowel obstruction with a “target sign” suggesting intussusception. Air enema failed to relieve symptoms. Preoperative ultrasonography was performed but did not identify the Meckel's diverticulum or internal hernia, highlighting the diagnostic limitations of ultrasound for this rare entity. Diagnostic laparoscopy through a single umbilical incision revealed an MDB entrapping 40 cm of ileum. The hernia was reduced laparoscopically, and the umbilical incision was extended to 20 mm for extracorporeal resection. Wedge resection of the Meckel's diverticulum with *en bloc* excision of the MDB origin was performed, followed by hand-sewn, single-layer, end-to-end anastomosis using 4-0 absorbable sutures. Notably, no wound protector was used during bowel exteriorization, as this was not standard practice in our institution at the time of this surgery. We acknowledge that this represents a technical deviation from current recommendations for single-incision laparoscopic surgery, given the increased risk of wound contamination and fascial trauma when handling edematous, obstructed bowel. Postoperatively, the patient recovered without wound infection or other complications. However, we recognize this omission as a limitation and have since adopted routine use of umbilical wound protectors (e.g., Alexis® Laparoscopic System) for all similar procedures. Operative time was 65 min with 5 mL blood loss. Pathology confirmed ectopic gastric mucosa. Oral intake resumed on postoperative day 3, and discharge was on day 7 (Figure 1A).

### Case 2

An 11-year-old boy presented with colicky pain and non-bilious vomiting. CT showed dilated small bowel with multiple air-fluid levels. He had been treated conservatively for similar symptoms one week earlier. Preoperative ultrasound, like in Case 1, failed to detect the MD or MDB, reinforcing the difficulty of preoperative diagnosis for this condition. Laparoscopy demonstrated an MDB and a 4-cm MD with incarcerated ileal loops. Wedge resection of the diverticulum and hand-sewn end-to-end anastomosis were performed extracorporeally through the extended umbilical incision without wound protection, consistent with our institutional practice during this early period. As with Case 1, we acknowledge the absence of a wound protector as a technical shortcoming that has since been corrected in our subsequent practice. Despite this omission, no wound infection or dehiscence occurred. Operative time was 90 min. The patient tolerated fluids on postoperative day 3 and solids on day 5, with discharge on day 8 (Figure 1B).

### Case 3

A 7-year-old girl complained of peri-umbilical pain for 2 days. Ultrasound suggested mesenteric lymphadenitis but, as with the previous cases, did not identify the MD or internal hernia. Because of persistent tenderness, diagnostic laparoscopy was performed. An internal hernia containing 25 cm of ileum was found. LATUM was executed in 50 min using the same technique: wedge resection with hand-sewn anastomosis, without wound protector (early institutional practice). Recovery was uneventful, and she was discharged on postoperative day 7 (Figure 1C).

### Overall outcomes

All procedures were completed without conversion or complications. Median operative time was 65 min (range: 50–90, IQR: 55–75), estimated blood loss was 10 mL (range: 5–15, IQR: 7.5–12.5), and time to first flatus was 24 h (range: 18–36, IQR: 20–30). Median follow-up was 16 months (13–20). All children remain asymptomatic with normal growth and diet.

We identified 16 reports encompassing 25 children (Table 1) (4–19). Based on our series of studies, 28 cases were analyzed. 9 cases (32.14%) used LATUM, 8 cases (28.57%) used open resection, and 11 cases (39.29%) did not describe the surgical approach.

**Table 1:** Review of published pediatric reports with mesodiverticular band.

PMID	Year	Journal	First author	CASE NO.	Surgical	References
5301782	1968	CANADIAN JOURNAL OF SURGERY	Seagram	1	Not described	(4)
3337631	1988	ARCHIVES OF PATHOLOGY & LABORATORY MEDICINE	Pfalzgraf	2	Not described	(5)
2743761	1989	Conn Med	Brophy	5	Not described.	(6)
8957607	1996	CLINICAL NUCLEAR MEDICINE	Hawkins	1	Open	(7)
10983393	2000	Wiadomosci Lekarskie	Janusz	1	Not described	(8)
17212886	2006	JSLS-Journal of the Society of Laparoendoscopic Surgeons	Prasad	1	LATUM	(9)
18082787	2008	AMERICAN JOURNAL OF EMERGENCY MEDICINE	Ko	2	Not described	(10)
21686941	2009	BMJ Case Rep	Shaaban	1	Open	(11)
22643517	2011	JSLS-Journal of the Society of Laparoendoscopic Surgeons	Mohiuddin	1	LATUM	(12)
23197558	2012	Journal of Medical Ultrasound	Sun	1	LATUM	(13)
26310428	2015	Pediatria Integral	Kunitsu	1	LATUM	(14)
29145243	2017	Medicine (Baltimore)	Bertozzi	2	Open	(15)
34106190	2021	Andes Pediatrica	Soto	3	Open	(16)
35775669	2022	Ulusal Travma ve Acil Cerrahi Dergisi-Turkish Journal of Trauma & Emergency Surgery	Şık	1	Open	(17)
38576141	2024	American Journal of Case Reports	Lai	1	LATUM	(19)
39759674	2024	Cureus	Adams	1	LATUM	(18)

## Discussion

Internal hernia through a MDB is a rare but distinct complication of MD (18). The embryology is well established: persistence of the vitelline artery leads to a tethering band that fixes the diverticulum to the mesentery, thereby creating a potential ring. Because the hernia orifice is small and the neck is vascular, bowel incarceration and subsequent strangulation can occur rapidly (12).

Pre-operative diagnosis remains challenging. Typical features of small-bowel obstruction are present, but the exact aetiology is seldom suspected. CT may show clustered, dilated loops and a “whirl” sign, yet these findings are non-specific (19, 20). In our series the correct diagnosis was made intra-operatively in every case. A low threshold for early diagnostic laparoscopy is therefore justified in any child with obstruction of unclear origin, especially when imaging is equivocal.

There are currently 25 cases (16 articles) of Meckel's diverticulum combined with intra-abdominal hernia reported in English literature. Both open surgery and LATUM are available surgical methods. Laparoscopy offers several advantages. First, it allows comprehensive visualisation of the entire small bowel and precise identification of the band. Second, reduction of the hernia can be achieved under direct vision with minimal manipulation, thereby reducing the risk of iatrogenic perforation. Third, simultaneous resection of the MD eliminates the possibility of future haemorrhage or intussusception. Our median operative time (65 min) and blood loss (10 mL) compare favourably with historical open controls. Moreover, the laparoscopic cohort had a significantly shorter hospital stay, a finding corroborated by the pooled literature data.

Among the 29 children treated by LATUM for MD-related internal hernia, the MDB was consistently found to originate from the diverticular tip and insert into the adjacent ileal mesentery, creating a 1–2 cm orifice through which 10–25 cm of ileum had herniated. Reduction was always accomplished laparoscopically; bowel viability was confirmed prior to exteriorisation. The umbilical port incision was extended to 15–20 mm and the affected bowel delivered through the umbilicus. Wedge resection of the MD together with the MDB origin was performed, followed by a hand-sewn, single-layer, end-to-end anastomosis using 4-0 or 5-0 absorbable sutures. The anastomosis was returned to the abdomen; the fascial defect was closed with figure-of-eight absorbable sutures. No patient required segmental ileal resection; no conversions to open laparotomy were reported.

Limitations of our study include its retrospective nature and small sample size. However, the rarity of the condition precludes large randomised trials. Multi-institutional registries are warranted to validate the advantages of LATUM. In addition, the use of a wound protector during umbilical exteriorization is now considered essential in LATUM, particularly for cases involving obstructed or edematous bowel. This device prevents wound contamination, protects fascial edges from traction trauma, facilitates specimen extraction, and reduces postoperative umbilical complications. We strongly recommend routine wound protection based on our experience and current best practices.

## Conclusion

LATUM is a safe, efficacious and cosmetically superior technique for children with MD complicated by internal hernia. A high index of suspicion and early laparoscopic exploration are essential to prevent bowel necrosis. We recommend LATUM as the first-line treatment in centres with paediatric minimally invasive expertise.

## Data Availability

The original contributions presented in the study are included in the article/Supplementary Material, further inquiries can be directed to the corresponding authors.
